# Unveiling the Therapeutic Potential of Suyin Detoxification Prescription in Diabetic Nephropathy: A Comprehensive Study Integrating Clinical Efficacy, Network Pharmacology, and Molecular Mechanisms

**DOI:** 10.1155/jdr/4566357

**Published:** 2025-12-22

**Authors:** Tuo Wei, Jiebo Huang, Bingying Wan, Bei Huang, Jing Cheng, Chao Shi, Chen Yong, Yan Li, Enchao Zhou

**Affiliations:** ^1^ Department of Nephrology, The First Clinical Medical College, Nanjing University of Chinese Medicine, Nanjing City, Jiangsu, China, njucm.edu.cn; ^2^ Department of Traditional Chinese Medicine, The First Affiliated Hospital of Wannan Medical College, Wuhu City, Anhui Province, China, wnmc.edu.cn; ^3^ Blood Purification Center of Nanjing Pukou District Hospital of Traditional Chinese Medicine, Nanjing City, Jiangsu, China; ^4^ Department of Nephrology, Changzhou Hospital of Chinese Medicine Affiliated to Nanjing University of Chinese Medicine, Changzhou City, Jiangsu, China; ^5^ The Personnel and Education Department of Ma’anshan City Hospital of Traditional Chinese Medicine, Ma’anshan City, Anhui Province, China; ^6^ Medical Oncology, The First Affiliated Hospital of Wannan Medical College, Wuhu City, Anhui Province, China, wnmc.edu.cn; ^7^ Key Laboratory of Tonifying Kidney and Anti-Aging, Jiangsu University, Nanjing City, Jiangsu Province, China, ujs.edu.cn

**Keywords:** diabetic nephropathy, FXR, inflammatory damage, network pharmacology, oxidative stress, SDP

## Abstract

**Background:**

Diabetic nephropathy (DN) is a severe complication of diabetes, characterized by progressive renal dysfunction and proteinuria. Suyin Detoxification Prescription (SDP), a traditional Chinese medicine (TCM) formulation, has shown promising clinical efficacy in alleviating DN symptoms. This study is aimed at comprehensively evaluating the therapeutic effects of SDP through clinical observations, network pharmacology analysis, and molecular mechanism exploration.

**Methods:**

This study employed a randomized controlled trial combined with network pharmacology, pathological analysis, immunofluorescence, and Western blot to comprehensively investigate the therapeutic efficacy and mechanisms of SDP in DN treatment.

**Results:**

Clinical data revealed that SDP significantly reduces serum creatinine levels (*p* = 0.009) and reduced proteinuria (*p* = 0.03) in DN patients. Network pharmacology successfully established an interaction network among active components of SDP, FXR targets, and DN pathological processes. Experimental validation demonstrated that the formula significantly downregulated TNF‐*α*, gp91, and P‐MLK1 expression in renal tissues of diabetic mice. Notably, FXR knockout markedly attenuated the renal protective effects of SDP in DN mice.

**Conclusion:**

SDP exerts renal protective effects in DN through FXR activation, mitigating oxidative stress and inflammatory damage, while reducing proteinuria and renal function impairment, providing novel therapeutic strategies for DN management.

## 1. Introduction

Diabetic nephropathy (DN) is one of the important complications of diabetic patients, which is mainly characterized by increased urinary protein and progressive decrease of glomerular filtration rate (GFR) [1, 2]. Recent studies have shown that about 30% of patients with T1DM and 20% of patients with T2DM will gradually develop DN, causing serious social burden [3, 4]. The rising prevalence of diabetes, driven by factors such as obesity, sedentary lifestyles, and aging populations, has contributed to the increased incidence of DN. In the United States, DN accounts for nearly half of all end‐stage renal disease cases, highlighting its public health impact [5, 6].

Pathophysiologically, chronic hyperglycemia promotes oxidative stress, inflammation, and advanced glycation end‐product accumulation, leading to damage in glomerular endothelial cells, podocytes, and tubular epithelial cells. Although emerging therapies such as SGLT2 inhibitors and GLP‐1 receptor agonists show promise, current treatments remain inadequate in addressing the multifaceted pathology of DN, underscoring the need for novel therapeutic strategies.

Traditional Chinese medicine (TCM) has garnered attention for its multitarget approach to DN management. Compounds containing herbs like *Astragalus membranaceus*, *Rheum palmatum*, and *Salvia miltiorrhiza* demonstrate renoprotective effects by modulating oxidative stress, inflammation, fibrosis, and autophagy [7–10]. Active components such as Astragaloside IV and emodin can inhibit the TGF‐*β*/Smad pathway, reduce podocyte injury, and ameliorate renal fibrosis. Furthermore, TCM formulations like Shenyan Kangfu Tablets and Tangshen Formula show synergistic effects in improving renal function [11–14]. Emerging research suggests that TCM may also regulate gut microbiota and mitochondrial function, offering new mechanistic insights. However, integrating TCM into modern frameworks requires stronger clinical and mechanistic validation [15, 16].

Among various TCM formulations, the Suyin Detoxification Prescription (SDP) has demonstrated significant potential in treating chronic kidney disease, particularly DN. Previous studies indicate that SDP possesses anti‐inflammatory, antioxidant, and antifibrotic activities. For instance, Ge et al. reported that SDP reduces trimethylamine N‐oxide (TMAO) levels by modulating gut microbiota, thereby inhibiting renal tubular ferroptosis and attenuating renal fibrosis [17]. Clinical observations suggest that SDP improves renal function and reduces proteinuria. Mechanistic studies have identified active components in SDP, such as those from *Perilla* leaf and *Artemisia capillaris*, which contain bioactive compounds like quercetin and capillarisin. These components modulate signaling pathways, including MAVS/NLRP3 and ERK/p38 MAPK, thereby reducing renal injury [18, 19]. It is important to note that the MAVS/NLRP3 pathway modulation is a finding associated with the whole SDP formulation rather than a single herb. However, research on SDP’s efficacy in DN remains limited. This study systematically evaluates the therapeutic effects and safety of SDP to provide evidence‐based support for its use in DN treatment, while potentially uncovering novel therapeutic targets.

## 2. Materials and Methods

### 2.1. The Composition of the Prescription of SDP

The SDP formulation comprises the following herbs: *Artemisia capillaris* (15 g), *Perilla* leaf (10 g), *Serissa japonica* (20 g), *Smilax glabra* (30 g), *Trogopterus* dung (10 g), *Carthamus tinctorius* (10 g), *Typha* pollen (10 g), raw *Astragalus* (30 g), *Rheum palmatum* (8 g), oyster shell (30 g), *Poria cocos* (10 g), and *Cornus officinalis* fruit (10 g). All herbal materials were sourced from GMP‐certified suppliers to ensure consistency. Standardized decoction protocols specifying water‐to‐herb ratios, extraction time, and temperature were followed, with quality control performed via marker compound quantification.

### 2.2. Patients’ Medical and Clinical Data

This study enrolled 50 patients with DN from the First Affiliated Hospital of Wannan Medical College, Jiangsu Provincial TCM Hospital, and Nanjing Pukou District TCM Hospital between February 2024 and February 2025. The trial protocol received prospective registration from the International Traditional Medicine Clinical Trials Registry (Registration Number ITMCTR2025000668). The ethical approval for the main study period was obtained (Approval No. 20250008), while the referenced approval number (2021NL‐182‐01) pertained to the preliminary exploratory phase of the research. All patients provided written informed consent.

Inclusion criteria were as follows: age 18–80 years, diagnosis of Type 2 diabetes per WHO criteria, DN stages G(2‐5)A(1‐3) per DNGA classification, and TCM syndrome differentiation of toxin‐stasis obstructing collaterals. Exclusion criteria included nondiabetic kidney disease, severe comorbidities (e.g., liver dysfunction, heart failure, stroke, and malignancy), recent metabolic disorders (e.g., diabetic ketoacidosis), urinary tract infections, or allergic constitution. Two patients dropped out after randomization: one in the treatment group due to voluntary withdrawal after symptom improvement and one in the control group lost to follow‐up, resulting in 24 patients per group completing the trial.

The patients were randomly divided into a control group receiving standard treatment (including glycemic control, blood pressure management with ACEI/ARB if tolerated, and lipid‐lowering therapy as needed) and a treatment group receiving standard treatment plus SDP. The formula, comprising 12 herbs, was prepared uniformly across centers with strict SOPs for decoction. Treatment duration was 6 weeks (three courses of 2 weeks each). Outcomes included TCM symptom scores (Table 1), renal function (e.g., eGFR and 24‐h proteinuria), and safety indicators (e.g., liver function). Efficacy was assessed per TCM symptom improvement criteria, with total efficacy calculated as the sum of cured, markedly effective, and effective cases.

**Table 1:** TCM syndrome rating scale.

**Symptom**	**0 points (none)**	**1 point (mild)**	**2 points (moderate)**	**3 points (severe)**
Low back pain	None	Occasional low back pain, does not affect daily activities	Frequent low back pain, barely able to perform daily activities	Severe low back pain, unable to perform daily activities
Fatigue	None	Unable to endure heavy labor, can perform light physical work	Feeling fatigued during normal activities, intermittent, barely able to perform daily activities	Feeling fatigued even at rest, persistent, unable to perform daily activities
Poor appetite and bloating	None	Mild reduction in food intake, mild bloating, appetite acceptable	Significant reduction in food intake, occasional bloating, poor appetite	Unable to eat, significant bloating, vomiting upon eating
Nausea and vomiting	None	Occasional nausea and vomiting	Frequent nausea and vomiting, few vomiting episodes	Frequent nausea and vomiting
Edema	None	Morning facial and/or limb swelling, slight indentation upon pressing	Significant facial and/or limb swelling, half‐finger indentation upon pressing	Severe facial and/or limb swelling, one‐finger indentation upon pressing
Dizziness	None	Occasional dizziness, does not affect daily activities	Frequent dizziness, no vertigo	Severe dizziness, may include vomiting, vertigo, unable to perform daily activities
Increased nocturia	None	Nocturia 1–2 times	Nocturia 3–4 times	Nocturia ≥ 5 times
Constipation	None	Hard and difficult bowel movements, once daily	Hard stools, bowel movements every 2–3 days	Hard stools, bowel movements less than once every 3 days

*Note:* The TCM syndrome score was assessed based on patient‐reported severity for each symptom.

### 2.3. Network Pharmacology Approach

This study systematically explored the mechanisms of SDP in treating DN. Active components from 12 herbs were screened using TCMSP (OB ≥ 30*%* and DL ≥ 0.18), and their targets were predicted via TCMSP and DrugBank. A drug–component–target network was constructed using Cytoscape 3.8.2. DN‐related targets from GeneCards were intersected with formula targets, and common genes were analyzed via GO and KEGG enrichment in DAVID. Results were visualized using the Venn diagrams, bar/bubble charts, and network graphs to elucidate multitarget mechanisms.

### 2.4. Animal Models and Treatment Protocols

Animal experiments were approved by the Nanjing Pukou District Hospital of Traditional Chinese Medicine Animal Ethics Committee (Approval No. 20210028) and complied with NIH guidelines. Forty C57BL/6 mice (25 wild type and 15 FXR knockout) were divided into eight groups (*n* = 5/group): (1) blank control (BC), (2) diabetes model (DM), (3) DM + dapagliflozin (DGLJ), (4) DM + SDP low dose (SDP(L)), (5) DM + SDP high dose (SDP(H)), (6) FXR^−/−^ + BC, (7) FXR^−/−^ + DM, and (8) FXR^−/−^ + DM + SDP(H). A diabetic model was established through five consecutive days of STZ (50 mg/kg) intraperitoneal injections; fasting blood glucose > 16.4 mmol/L confirmed successful modeling. Mice were fed a high‐fat diet (21% fat, 50% carbohydrates, and 1.5% cholesterol) for 16 weeks.

The animal doses of SDP were quantitatively converted based on body surface area equivalence from the human clinical dose. The low dose (45 g/60 kg/day) and high dose (90 g/60 kg/day) correspond to 1× and 2× the clinical equivalent dose, respectively. This dose design was referenced from previous pharmacodynamic studies on SDP granules to ensure comparability, although the present study used a traditional water decoction. After 4 weeks of treatment, blood and kidney tissues were collected for analysis.

### 2.5. Blood and Urine Biochemistry

The animals were placed in metabolic cages for 24 h to collect urine samples. Urinary protein concentrations were quantified using a BIOSTEC protein analyzer (BA400, Spain), while renal function was evaluated by serum biochemical testing performed on a Hitachi automated analyzer (7180, Japan).

### 2.6. Reagents

The following primary antibodies were used: anti‐FXR (Proteintech, Cat# 25055‐1‐AP, 1:1000), anti‐gp91 (BD Biosciences, Cat# 611414, 1:500), anti‐p‐MLKL (Cell Signaling Technology [CST], Cat# 18640S, 1:1000), and anti‐TNF‐*α* (Proteintech, Cat# AB205587, 1:800). Corresponding horseradish peroxidase (HRP)–conjugated secondary antibodies included anti‐mouse IgG (Proteintech, Cat# SA00001‐1) and anti‐rabbit IgG (Proteintech, Cat# SA00001‐2), both used at a dilution of 1:5000.

### 2.7. Western Blot Analysis

Kidney tissues were homogenized in lysis buffer with protease/phosphatase inhibitors (Beyotime, China). Proteins were denatured, separated by 10% SDS‐PAGE (Bio‐Rad, China), and transferred to PVDF membranes (Millipore, United States). Membranes were blocked, incubated with primary (12 h, 4°C) and secondary antibodies (1 h, RT), and visualized using chemiluminescence (ChemiDoc XRS, Bio‐Rad). Quantification was performed using ImageJ (1.52a).

### 2.8. Histopathology

Renal tissues were preserved in 4% paraformaldehyde, processed through graded dehydration, and subsequently stained with H&E (Servicebio, GP1031), Masson’s trichrome (Servicebio, GP1032), and PAS (Servicebio, GP1039). Morphometric analysis of stained sections was performed using ImageJ software for quantitative assessment.

### 2.9. Immunofluorescence

Renal sections (40 *μ*m) were stained with anti‐FXR and anti‐gp91 antibodies. Images were captured using a slide scanner (3DHISTECH, no. Pannoramic MIDI) and analyzed with ImageJ to quantify antibody expression as integrated density.

### 2.10. Statistical Data Evaluation

Statistical analysis was conducted using GraphPad Prism 9.5. Continuous data were tested for normality; normally distributed data with equal variances were analyzed by *t*‐test (two groups) or ANOVA (multiple groups), while nonnormal data were analyzed using nonparametric tests. Categorical data, expressed as percentages or counts, were analyzed by chi‐square or Fisher’s exact test. Significance levels were set at *p* > 0.05 (nonsignificant), *p* < 0.05 (significant), and *p* < 0.01 (highly significant).

## 3. Results

### 3.1. Clinical Study on the Efficacy of SDP in Treating DN

#### 3.1.1. Baseline Characteristics and Comparative Analysis of Age, Gender, and Blood Pressure Between Treatment and Control Groups

The baseline characteristics of participants were comparable between the treatment and control groups. The mean age was 63.38 ± 9.79 years in the treatment group and 65.71 ± 9.96 years in the control group (*p* > 0.05). Gender distribution showed 8 females and 17 males in the treatment group and 10 females and 15 males in the control group (*p* > 0.05). Mean systolic blood pressure (SBP) was 139.3 ± 18.42 mmHg (treatment) versus 140.4 ± 20.8 mmHg (control), and mean diastolic blood pressure (DBP) was 72.8 ± 10.95 mmHg (treatment) versus 73.4 ± 8.37 mmHg (control), with no significant differences (*p* > 0.05). These results confirm balanced baseline characteristics between groups. Detailed data are presented in Table 2.

**Table 2:** Comparison of age, gender, and blood pressure between the two groups.

**Variable**	**Treatment group (** **n** = 25**)**	**Control group (** **n** = 25**)**	**p** **value**
Age (years)	63.38 ± 9.79	65.71 ± 9.96	0.477
Gender			
Female	8	10	0.56
Male	17	15	0.56
Blood pressure			
SBP (mmHg)	139.3 ± 18.42	140.4 ± 20.8	0.874
DBP (mmHg)	72.8 ± 10.95	73.4 ± 8.37	0.941

#### 3.1.2. Efficacy and Safety of Treatment on Renal Function, Urinary Protein, and TCM Syndrome Scores in Patients

The analysis of serum creatinine and eGFR levels revealed significant improvements in the treatment group, with a decrease in serum creatinine (*p* = 0.009) and an increase in eGFR (*p* = 0.004) after treatment, while the control group showed a significant increase in serum creatinine (*p* = 0.028) and a decrease in eGFR (*p* = 0.012), indicating renal function deterioration. No significant changes were observed in blood urea nitrogen or uric acid levels within or between groups (*p* > 0.05), suggesting no impact on these parameters. Blood glucose levels remained stable in both groups (*p* > 0.05), but the treatment group demonstrated a significant reduction in 24‐h urinary protein levels (*p* = 0.03), highlighting a potential renal protective effect. Detailed results are presented in Table 3.

**Table 3:** Comparison of clinical parameters before and after treatment between the treatment and control groups (data are presented as mean ± standard deviation).

**Parameter**	**Treatment group (** **n** = 24**)**	**Control group (** **n** = 24**)**	**p** **(between groups)**
Serum creatinine (*μ*mol/L)			
Before treatment	206.1 ± 100.1	266.9 ± 145.3	0.0982
After treatment	182.2 ± 81.1^#^	306.1 ± 166.3^#△^	0.002
*p* (within group)	0.009	0.028	
eGFR (mL/min/1.73 m^2^)			
Before treatment	34.69 ± 17.14	26.78 ± 9.66	0.054
After treatment	40.70 ± 10.80^#^	22.60 ± 10.80^#△^	0.001
*p* (within group)	0.004	0.012	
Blood urea nitrogen (mmol/L)			
Before treatment	13.74 ± 4.87	13.76 ± 4.98	0.490
After treatment	14.89 ± 6.49	16.47 ± 8.53	0.251
*p* (within group)	0.975	0.184	
Uric acid (*μ*mol/L)			
Before treatment	391 ± 104.3	385.7 ± 106	0.859
After treatment	360 ± 69.31	379.8 ± 83.60	0.831
*p* (within group)	0.056	0.376	
Blood glucose (mmol/L)			
Before treatment	7.47 ± 2.0	7.64 ± 4.39	0.866
After treatment	6.76 ± 1.53	5.92 ± 2.50	0.164
*p* (within group)	0.125	0.173	
24‐h urine protein (g/24 h)			
Before treatment	1.94 ± 1.42	1.85 ± 1.77	0.800
After treatment	1.47 ± 1.00^#^	1.74 ± 1.20	0.301
*p* (within group)	0.030	0.580	

^#^Significant within‐group difference from baseline (*p* < 0.05).

^△^Significant between‐group difference at the same time point (*p* < 0.05).

#### 3.1.3. Changes in TCM Syndrome Scores and Efficacy Evaluation

Paired and independent *t*‐tests revealed a significant reduction in TCM syndrome scores in the treatment group after 6 weeks (*p* = 0.001), with no significant change in the control group (*p* = 0.093). The improvement was significantly greater in the treatment group (*p* = 0.037). Consistent with the improvement in TCM syndrome scores, Fisher’s exact test showed a higher total effective rate in the treatment group (87.5%) compared to the control group (37.5%, *p* < 0.05), indicating superior clinical efficacy. Detailed results are presented in Table 4.

**Table 4:** Comparison of TCM syndrome scores and clinical efficacy between the treatment and control groups (data are presented as mean ± SD).

**Parameter**	**Treatment group (** **n** = 24**)**	**Control group (** **n** = 24**)**	**p** **(between groups)**
TCM syndrome score			
Before treatment	9.17 ± 2.75	8.75 ± 2.74	0.602
After treatment	6.33 ± 2.32^#^	7.71 ± 2.12^△^	0.037
*p* (within group)	0.001	0.093	
Clinical efficacy			
Recovery	0 (0%)	0 (0%)	—
Marked improvement	9 (37.5%)	3 (12.5%)	—
Improvement	12 (50%)	6 (25%)	—
No improvement	3 (12.5%)	15 (62.5%)^△^	—
Total effective rate	21 (87.5%)	9 (37.5%)^△^	—

^#^Significant pre‐post difference within group (*p* < 0.05).

^△^Significant intergroup difference at the same timepoint (*p* < 0.05).

#### 3.1.4. Safety Evaluation

One patient in the treatment group dropped out because his symptoms improved and he refused to continue treatment. One patient in the control group was lost to contact without explanation and dropped out of the group. Twenty‐four patients in each group completed the clinical trial. All the remaining patients underwent comprehensive vital signs and liver/kidney function monitoring, with no severe adverse events (e.g., infections, heart failure, liver failure, or bleeding) reported in either group. In the treatment group, one patient developed a rash, which resolved with loratadine, and another had a transient AST elevation that normalized after dietary adjustments and rest. All other patients reported no discomfort, and adverse events were mild, with no impact on trial progress.

### 3.2. Renal Protective Effects of SDP in Diabetic Mice

To investigate the renal protective effects of SDP in diabetic mice, we administered high‐ and low‐dose treatments via gavage. The results demonstrated that diabetic model mice exhibited significant weight loss (Figure 1a) (*p* < 0.01). SDP intervention effectively reduced these abnormalities. Furthermore, SDP significantly alleviated proteinuria, with both high‐ and low‐dose groups showing decreased 24‐h urinary protein levels compared to the model group (*p* < 0.05), exhibiting a dose‐dependent effect (Figure 1b). The high‐dose group also demonstrated a mild hypoglycemic effect (Figure 1c). While SDP had no significant impact on blood urea nitrogen (Figure 1d), it notably reduced blood creatinine (*p* < 0.01, Figure 1e) and blood uric acid levels (*p* < 0.05, Figure 1f). The high‐dose SDP group demonstrated a more pronounced effect in lowering serum creatinine.

Figure 1SDP ameliorates renal function and pathology in diabetic mice. (a) Body weight. (b) 24‐h urinary protein quantification. (c) Blood sugar level. (d) Blood urea nitrogen. (e) Serum creatinine. (f) Blood uric acid. (g) HE, PAS, and Masson staining. (h) Masson staining intensity analysis. Results are expressed as mean ± standard deviation (*n* = 5). Statistical significance is denoted as follows:  ^∗^ *p* < 0.05,  ^∗∗^ *p* < 0.01,  ^∗∗∗^ *p* < 0.001, and  ^∗∗∗∗^ *p* < 0.0001; ns indicates nonsignificant differences.

**Figure:**
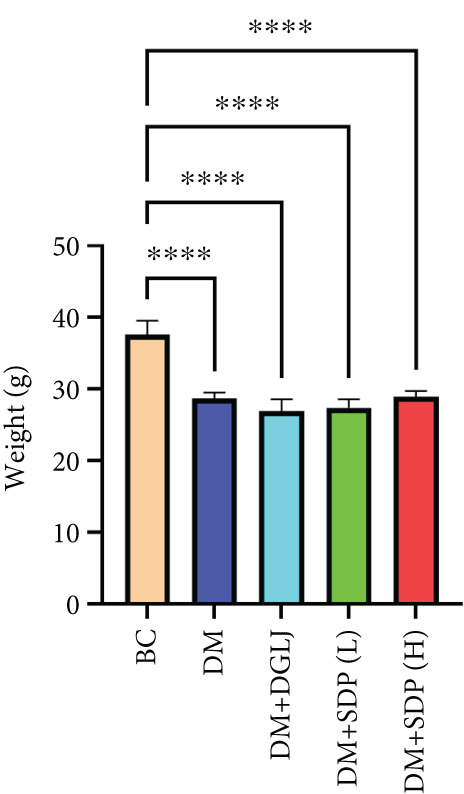
(a)

**Figure:**
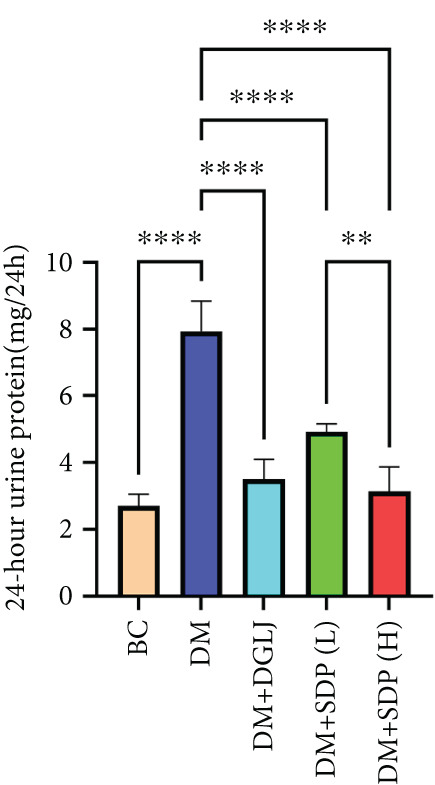
(b)

**Figure:**
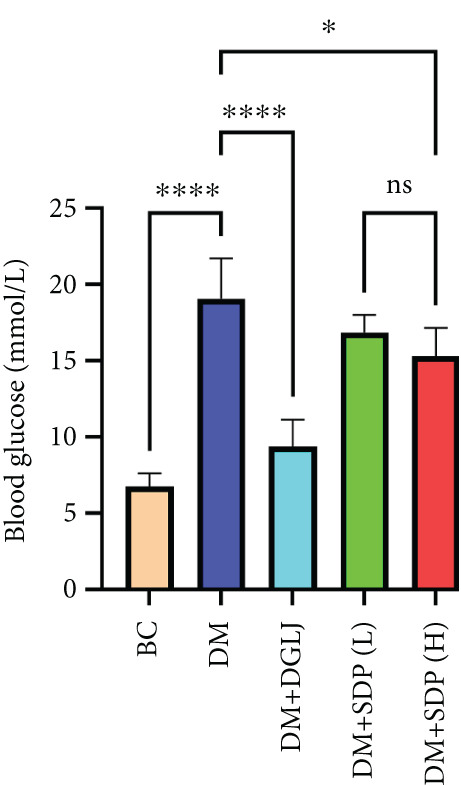
(c)

**Figure:**
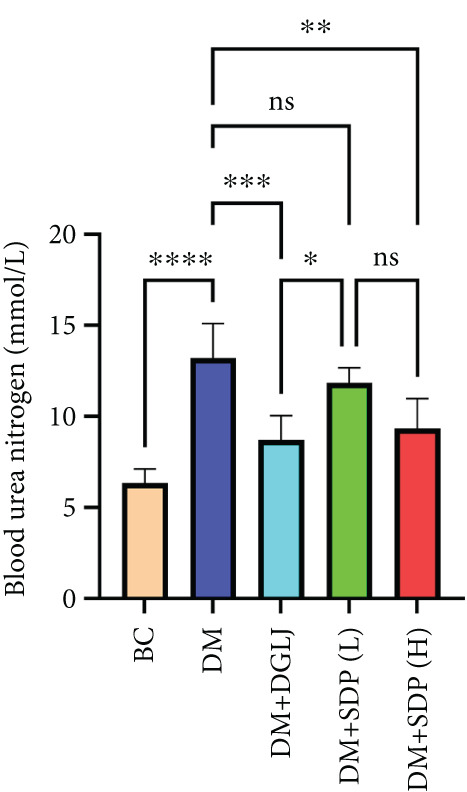
(d)

**Figure:**
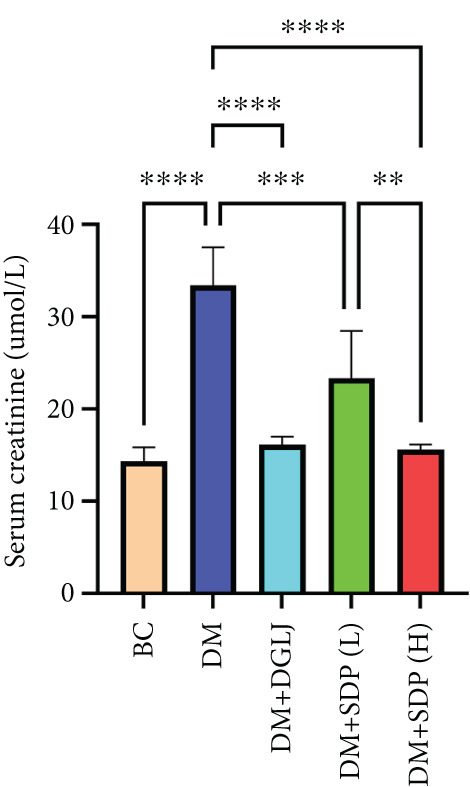
(e)

**Figure:**
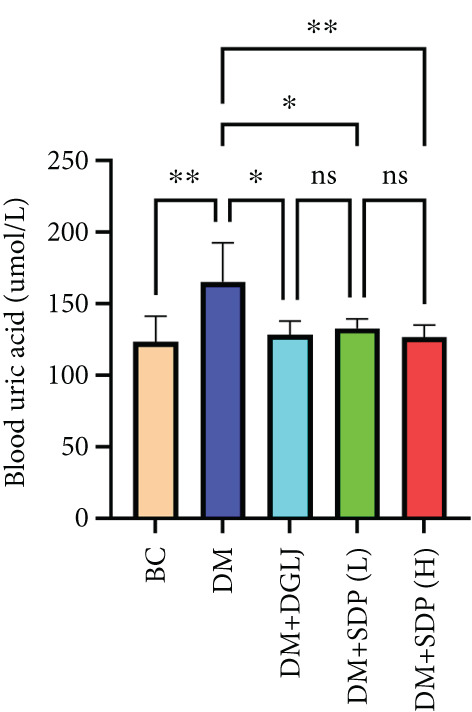
(f)

**Figure:**
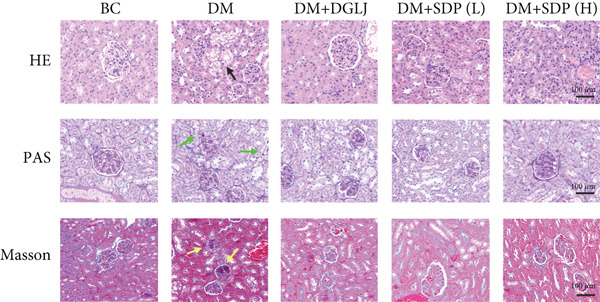
(g)

**Figure:**
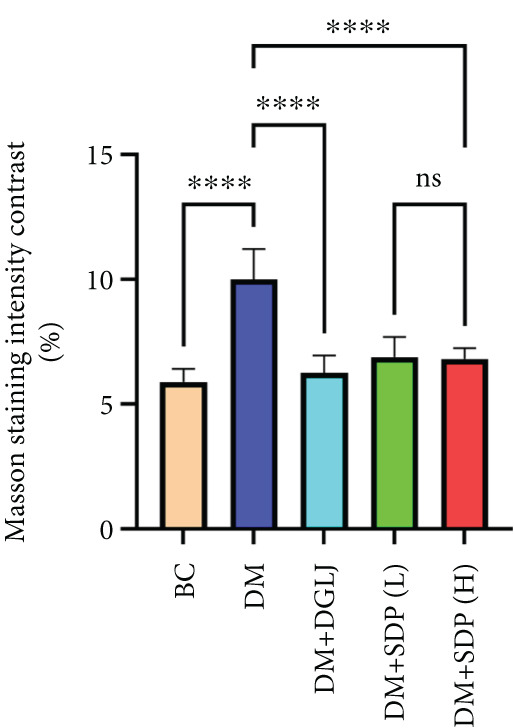
(h)

To further assess renal protection, we evaluated pathological changes in kidney tissues using HE, PAS, and Masson staining. SDP alleviated renal tubular vacuolization and casts formation (black and green arrows in Figure 1g). Masson staining revealed substantial collagen deposition in the DM group, whereas both DM + SDP(L) and DM + SDP(H) groups exhibited reduced blue‐stained collagen fiber areas after 4 weeks of intervention (yellow arrows in Figure 1g,h), confirming SDP’s efficacy in mitigating renal fibrosis progression.

### 3.3. A Network Pharmacology‐Based Model Was Constructed to Elucidate the Therapeutic Mechanisms of the SDP in Treating DN

The active ingredients of 12 herbal components from SDP were systematically screened using the TCMSP database with strict pharmacokinetic criteria (OB ≥ 30*%* and DL ≥ 0.18), identifying 107 bioactive compounds including 19 from *Perilla* leaf, 13 from *Artemisia capillaris*, 15 from *Poria*, 16 from raw *Astragalus*, 20 from *Cornus*, 8 from *Pollen Typhae*, and 16 from Rhubarb (Figure 2a). Target prediction through TCMSP and DrugBank databases yielded 483 unique potential targets, which were visualized in a drug–component–target network using Cytoscape 3.8.2, revealing *Cornus*, *Carthamus*, *Pollen Typhae*, and Rhubarb as key components with extensive target interactions. Comparative analysis with 8304 DN‐related targets from GeneCards identified 351 overlapping genes (Figure 2b), suggesting their potential therapeutic relevance. Functional enrichment analysis of these overlapping targets through DAVID demonstrated significant involvement in biological processes (inflammatory and metabolic regulation), cellular components (membrane and organelle systems), and molecular functions (protein binding and catalytic activity) (Figure 2c). KEGG pathway analysis further highlighted the Top 10 enriched pathways (Figure 2d), including the FXR‐bile acid signaling pathway—a critical regulator of metabolic homeostasis and inflammation in DN. These findings implicate multitarget regulatory mechanisms of SDP in DN treatment, particularly through FXR‐mediated modulation of bile acid metabolism and its downstream anti‐inflammatory effects. The integrated network pharmacology approach provides a comprehensive framework for understanding SDP’s therapeutic potential, with FXR activation emerging as a central node connecting multiple pathological processes in DN.

Figure 2Network pharmacology identifies FXR activation as a core mechanism of SDP against diabetic nephropathy. (a) Network diagram of the effective active component–target interactions of the SDP. (b) Venn diagram. (c) GO enrichment pathway. (d) KEGG enrichment pathway.

**Figure:**
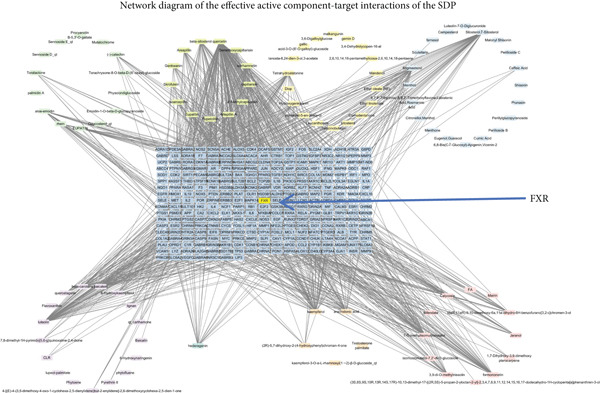
(a)

**Figure:**
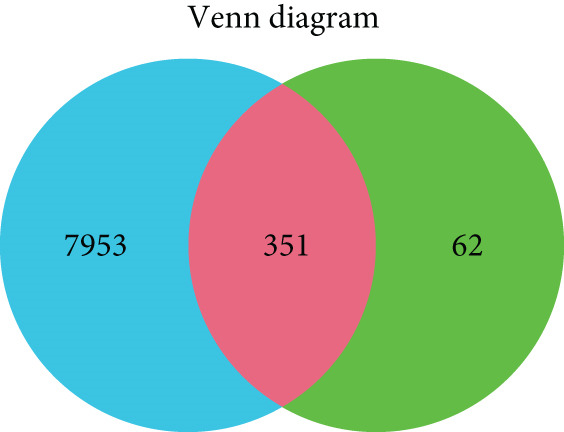
(b)

**Figure:**
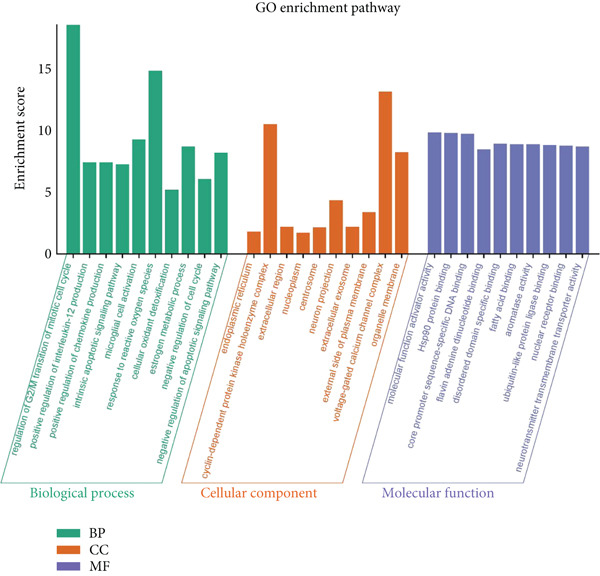
(c)

**Figure:**
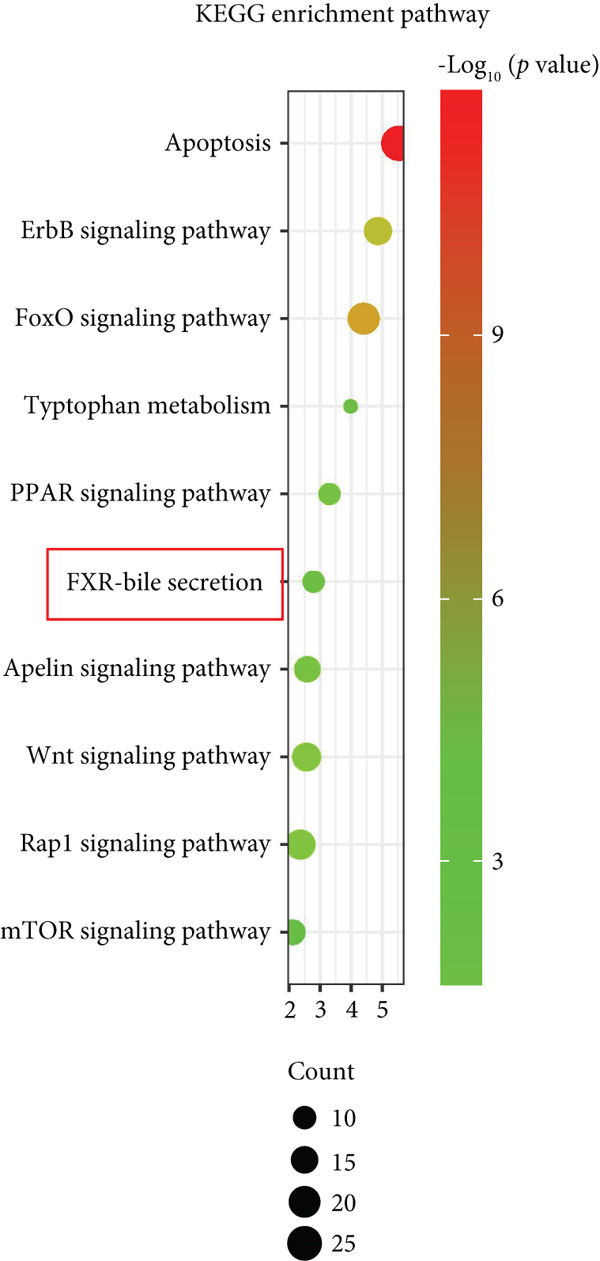
(d)

### 3.4. SDP Attenuates the Inflammation and Oxidative Stress in DN via FXR‐Mediated Pathway

To elucidate the molecular mechanisms underlying SDP’s renoprotective effects, we focused on the FXR‐mediated pathway, a key regulator of metabolic and inflammatory responses in DN. Immunofluorescence staining of FXR and gp91, along with WB analysis of serum FXR, TNF‐*α*, gp91, and P‐MLK1 expression levels, revealed that SDP(H) significantly upregulated FXR expression (Figure 3a,b) while suppressing gp91 (Figure 3c,d). WB results further indicated that SDP markedly reduced TNF‐*α*, gp91, and P‐MLK1 expression, with the high‐dose group demonstrating superior efficacy (Figures 3e, 3f, 3g, and 3h), and concurrently increased FXR levels (Figure 3e,i). Collectively, these findings suggest that SDP exerts its pharmacological effects by upregulating FXR expression, thereby inhibiting oxidative stress (via gp91 downregulation) and inflammatory responses (via TNF‐*α* and P‐MLK1 suppression) in DN mice.

Figure 3SDP activates FXR to attenuate oxidative stress and inflammation in diabetic kidneys. (a) Immunofluorescence staining of FXR. (b) FXR fluorescence intensity. (c) Immunofluorescence staining of gp91. (d) gp91 fluorescence intensity. (e) Western blot analysis of P‐MLK1, gp91, TNF‐*α*, and FXR expression. (f) Protein expression levels of P‐MLK1. (g) Protein expression levels of gp91. (h) Protein expression levels of TNF‐*α*. (i) Protein expression levels of FXR. Values represent mean ± SD (*n* = 5/group), with significance levels denoted by asterisks ( ^∗^ *p* < 0.05,  ^∗∗^ *p* < 0.01,  ^∗∗∗^ *p* < 0.001, and  ^∗∗∗∗^ *p* < 0.0001) and ns indicating nonsignificance.

**Figure:**
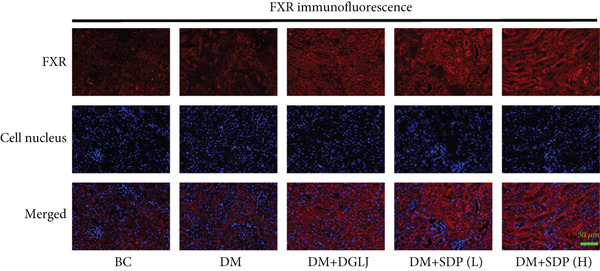
(a)

**Figure:**
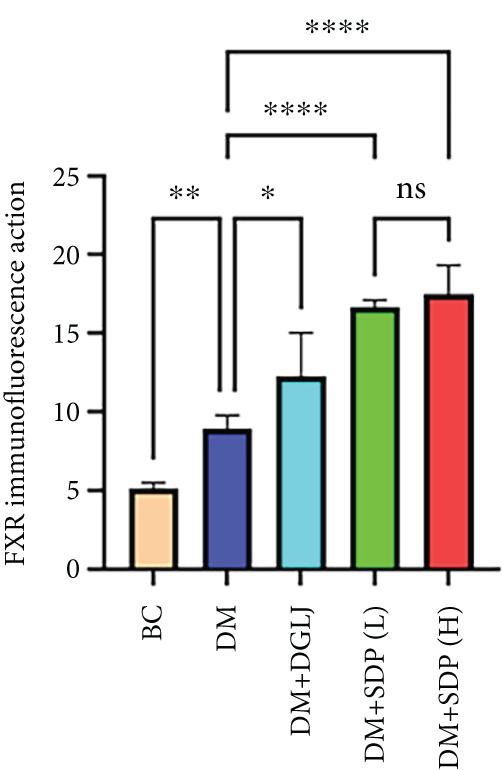
(b)

**Figure:**
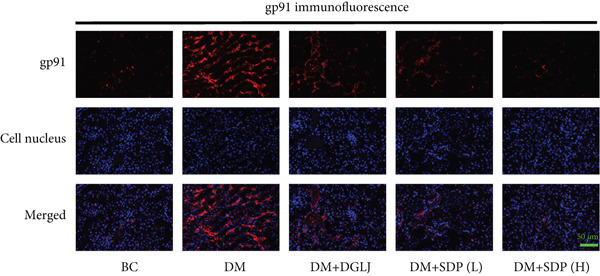
(c)

**Figure:**
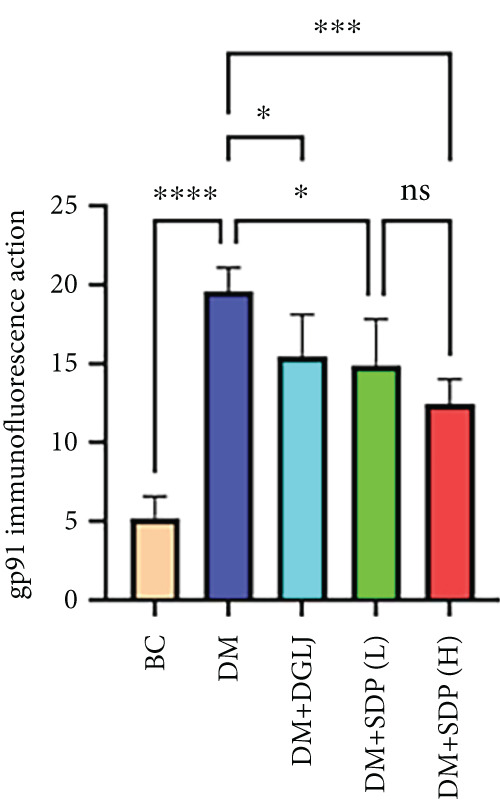
(d)

**Figure:**
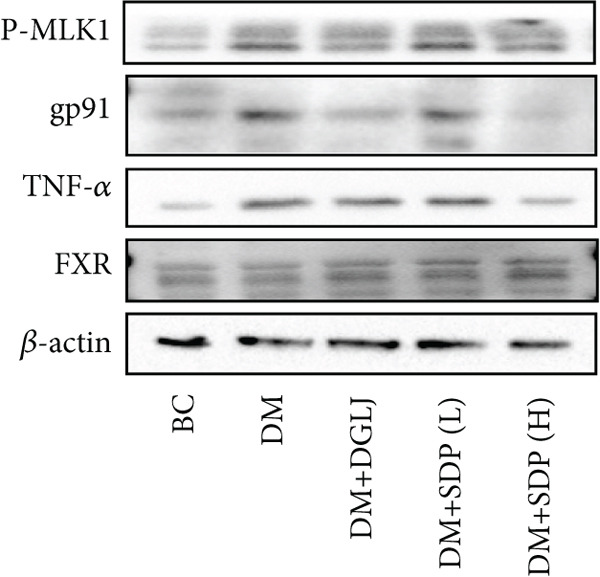
(e)

**Figure:**
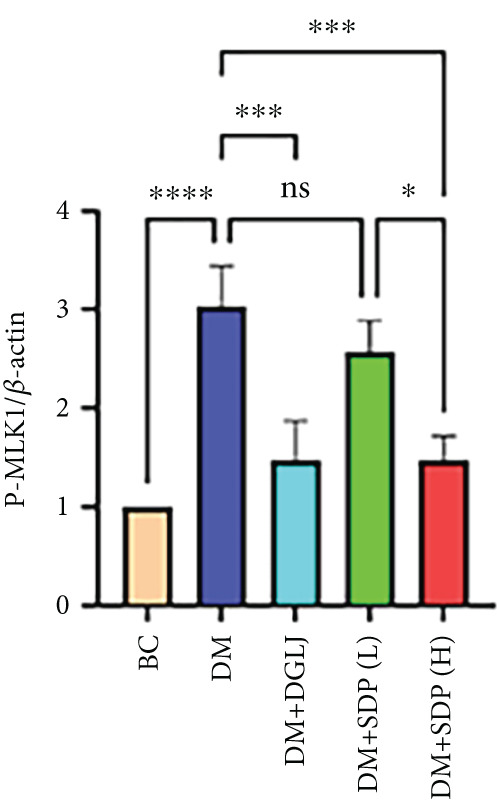
(f)

**Figure:**
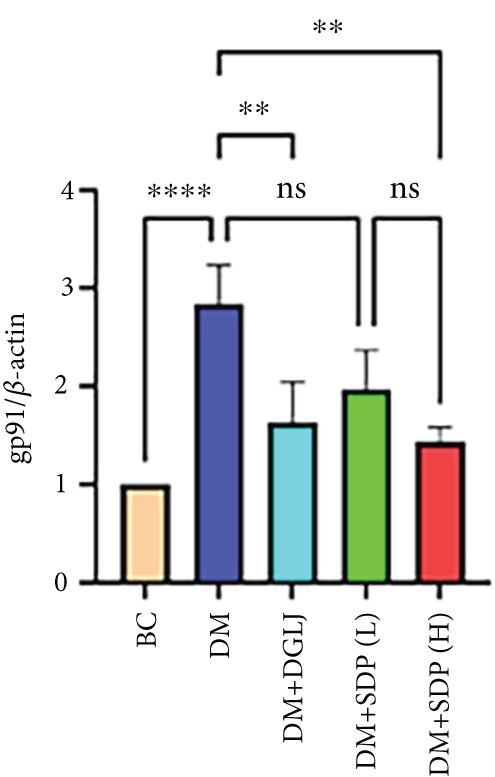
(g)

**Figure:**
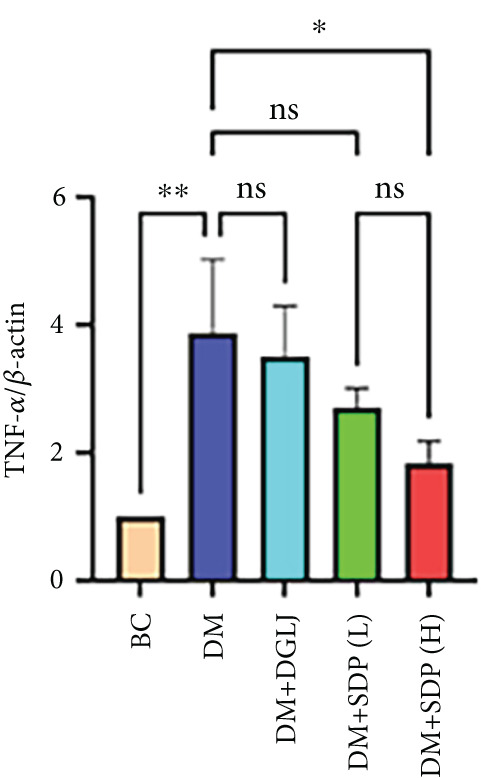
(h)

**Figure:**
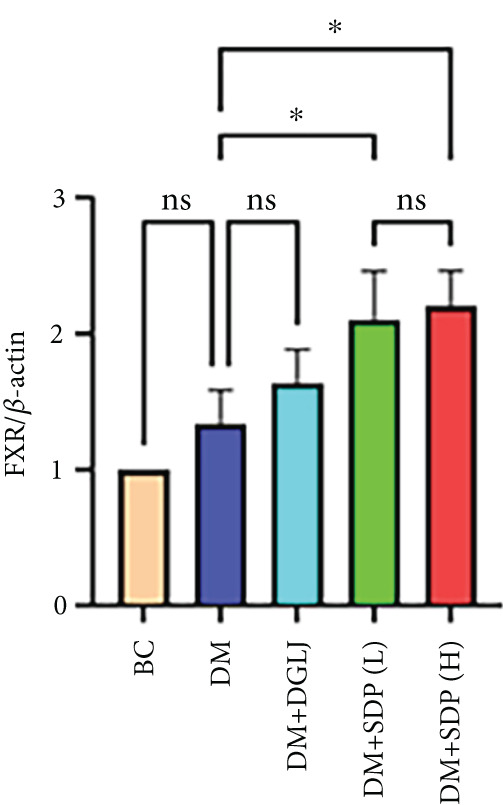
(i)

### 3.5. FXR as a Key Molecular Target of SDP in DN: Evidence From FXR Knockout Mouse Model

Our previous studies demonstrated that SDP significantly reduces 24‐h urinary protein and serum creatinine levels while alleviating oxidative stress in DN mice. However, its specific molecular targets remained unclear. Based on the formula’s composition and prior research, we hypothesized that FXR might be a potential target. To test this, we employed an FXR knockout mouse model. Results showed that the therapeutic effects of SDP were markedly attenuated in FXR knockout mice, including reduced efficacy in lowering 24‐h urinary protein (Figure 4a), and impaired improvement in renal function markers (Figures 4b, 4c, and 4d). HE, PAS, and Masson staining showed that SDP(H) did not significantly ameliorate renal injury in diabetic mice after FXR knockout (Figure 4e,h). Immunofluorescence and Western blot analyses further confirmed that the expression levels of gp91 and TNF‐*α* were not significantly different between the high‐dose and model groups in FXR knockout mice (Figures 4f, 4g, 4i, 4j, and 4k). These findings strongly suggest that FXR is a critical molecular target for the renal protective effects of SDP, providing a theoretical foundation for developing FXR‐based therapeutic strategies for DN.

Figure 4FXR knockout attenuates the renal protective effects of SDP in diabetic mice. (a) 24‐h urinary protein. (b) Blood urea nitrogen. (c) Serum creatinine. (d) Blood uric acid. (e) HE, PAS, and Masson staining. (f) Immunofluorescence staining of gp91. (g) Western blot analysis of TNF‐*α* and gp91. (h) Masson staining intensity analysis. (i) Immunofluorescence staining of gp91. (j) Protein expression levels of TNF‐*α*. (k) Protein expression levels of gp91. All quantitative data are shown as mean ± SD (*n* = 5 for each experimental group), where asterisks indicate statistical significance levels ( ^∗^ *p* < 0.05,  ^∗∗^ *p* < 0.01,  ^∗∗∗^ *p* < 0.001, and  ^∗∗∗∗^ *p* < 0.0001) and ns denotes statistically nonsignificant results.

**Figure:**
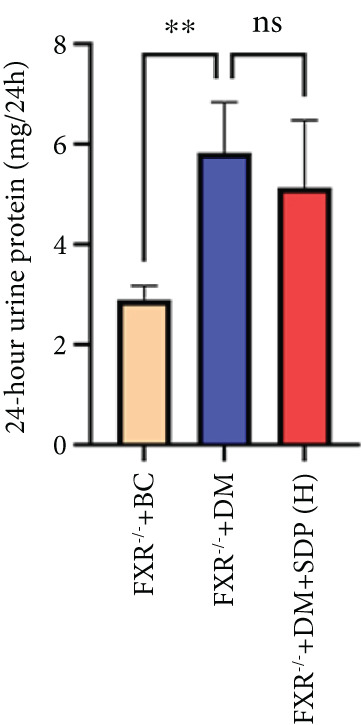
(a)

**Figure:**
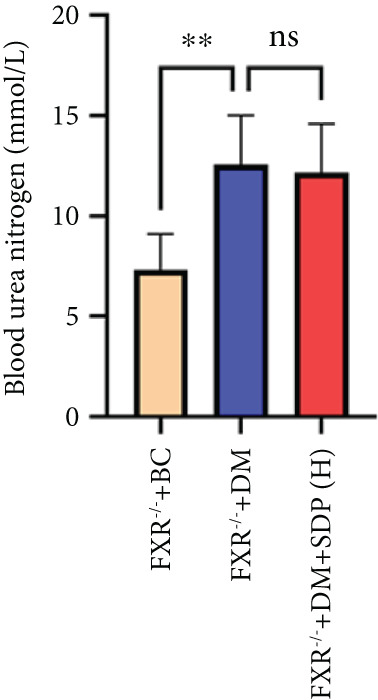
(b)

**Figure:**
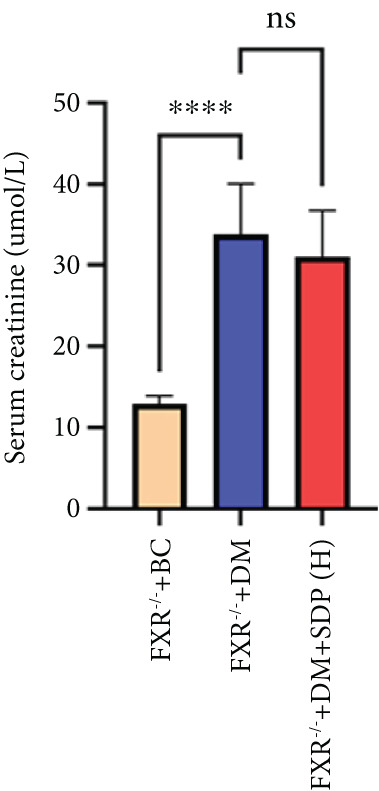
(c)

**Figure:**
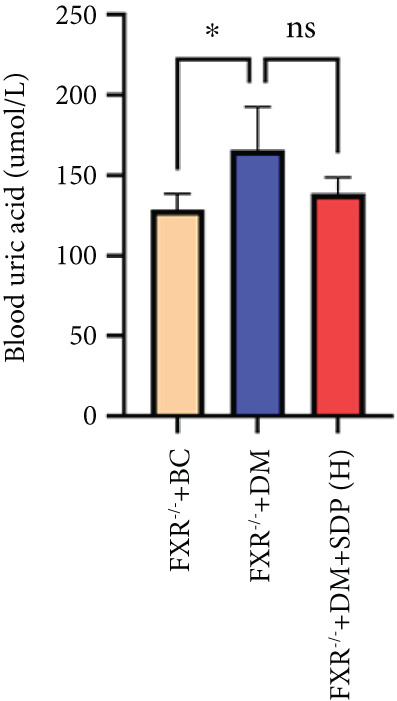
(d)

**Figure:**
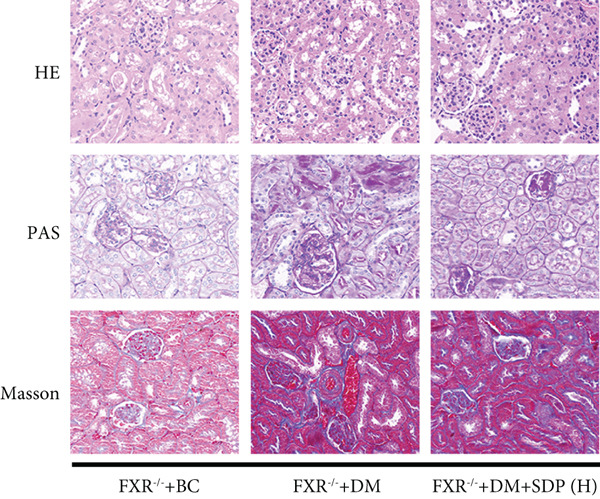
(e)

**Figure:**
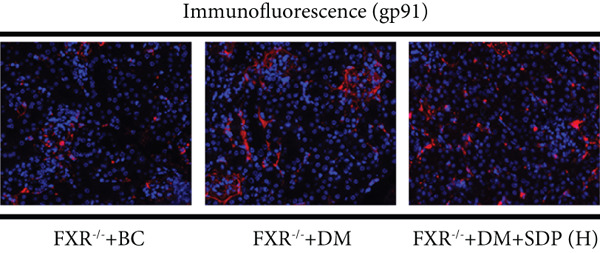
(f)

**Figure:**
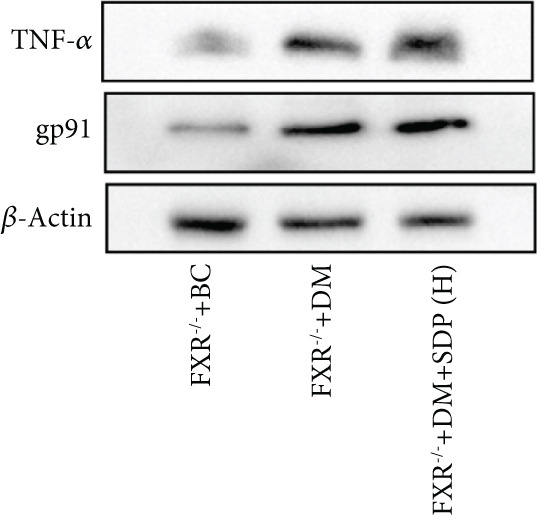
(g)

**Figure:**
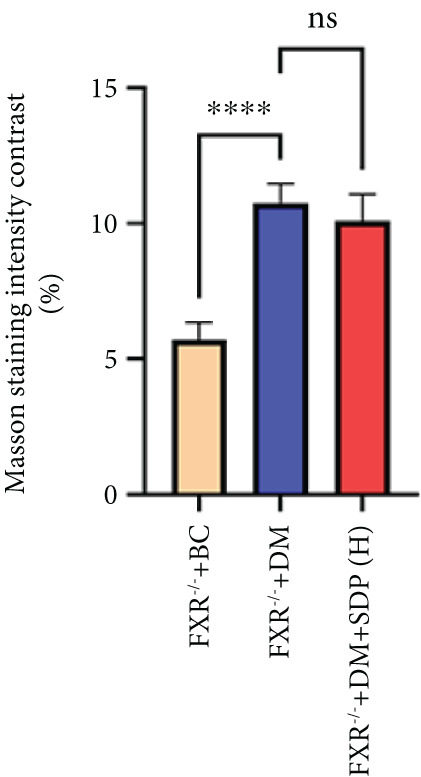
(h)

**Figure:**
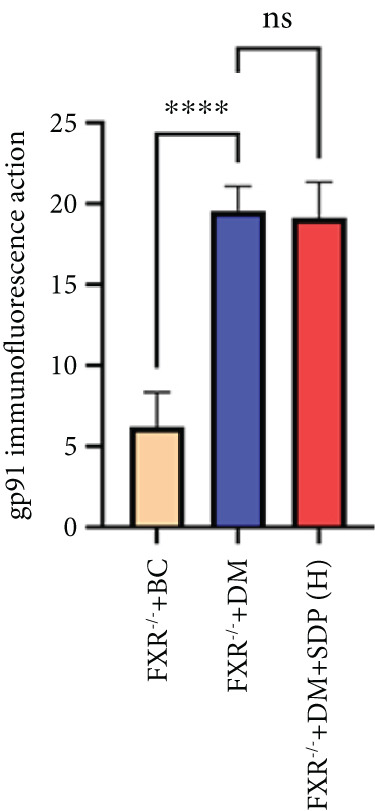
(i)

**Figure:**
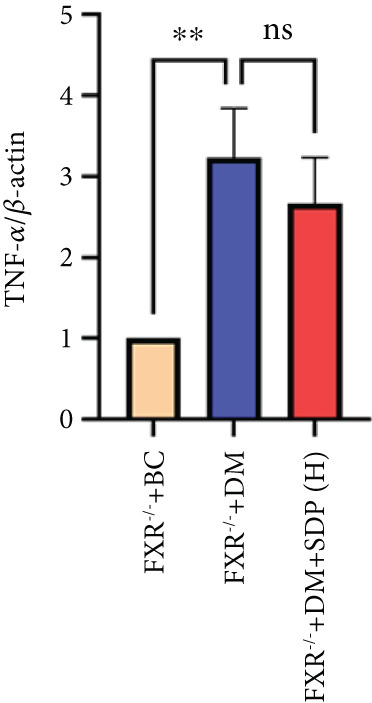
(j)

**Figure:**
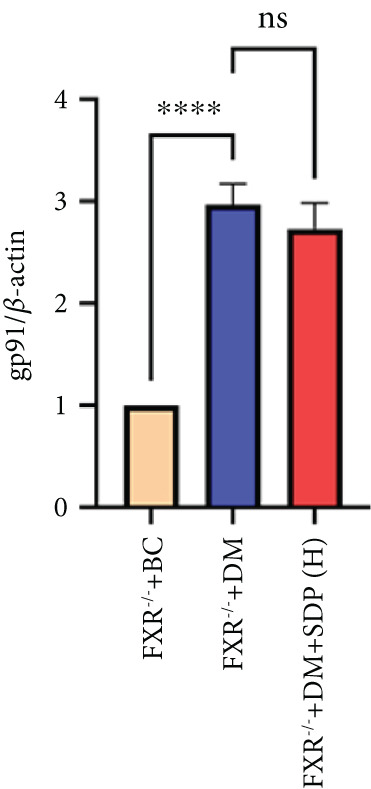
(k)

## 4. Discussion

The present study provides a comprehensive evaluation of the therapeutic potential of SDP in DN, integrating clinical efficacy, network pharmacology, and mechanistic insights. Our findings demonstrate that SDP significantly improves renal function by reducing proteinuria and serum creatinine, while activating the FXR pathway, a key regulator of metabolic and inflammatory responses in DN [20]. These results align with recent advancements emphasizing multitarget therapies and the gut–kidney axis in disease progression [21].

This study demonstrates that SDP significantly improves renal function in DN patients, effectively reducing Scr levels, increasing the eGFR, and decreasing 24‐h urinary protein excretion. Additionally, it alleviates TCM syndrome manifestations, with an overall efficacy rate of 87.5%. The decline in Scr suggests partial renal functional recovery. The lack of significant reduction in BUN and UA levels, however, may indicate that SDP’s restorative effect on tubular secretion function is incomplete, or that these parameters are influenced by other factors such as dietary protein/purine intake. Therefore, while SDP demonstrates clear benefits, an integrated management approach—including regular renal function monitoring and dietary modifications—remains essential for optimal DN patient outcomes.

Our network analysis revealed 351 shared targets between SDP and DN pathogenesis. Network pharmacology predicts that the SDP may exert anti‐inflammatory, antioxidant, and antifibrotic effects by modulating signaling pathways such as NF‐*κ*B and FXR. These predictions provide a theoretical foundation for further experimental validation. Network pharmacology studies have revealed that the SDP, composed of 12 traditional Chinese herbs, is primarily dominated by active components from *Perilla* leaf, *Artemisia capillaris*, raw *Astragalus*, *Typha* pollen, and *Rheum palmatum*, which exhibit a higher number of bioactive compounds and potential targets. For instance, *Perilla* leaf may alleviate renal injury by modulating inflammation and oxidative stress–related targets [22, 23]. *Artemisia capillaris* could protect renal function by improving lipid metabolism and inhibiting fibrosis [24]. Raw *Astragalus* may delay the progression of DN through enhanced immunomodulation and antioxidant activity [25]. Cattail pollen might mitigate glomerular damage by improving microcirculation and suppressing platelet aggregation [26]. Rhubarb could ameliorate renal function by regulating gut microbiota and reducing toxin accumulation [27]. These components likely exert synergistic therapeutic effects against DN‐induced renal injury through multitarget and multipathway mechanisms.

**Figure 5:**
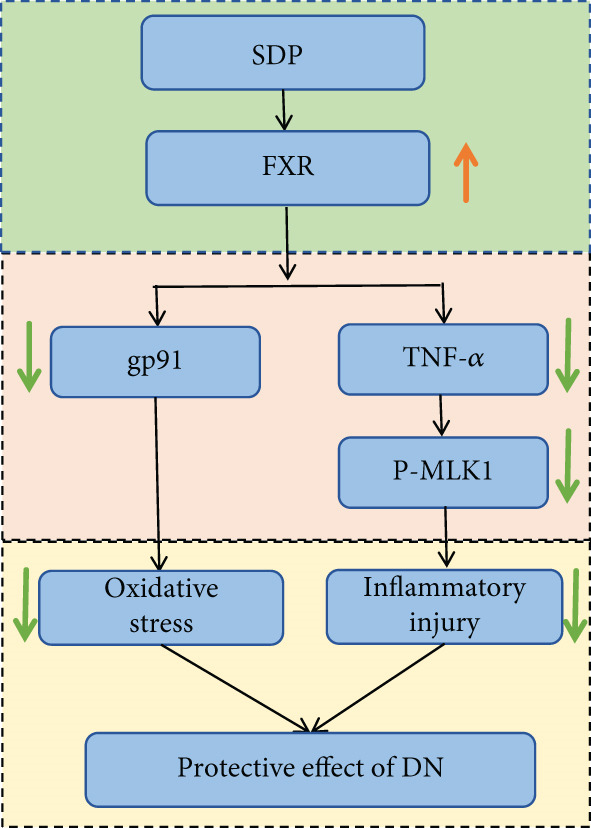
Mechanism of SDP attenuating renal injury in DN.

Oxidative stress plays a pivotal role in the pathogenesis of DN [28]. In this study, SDP intervention for 4 weeks dose‐dependently improved renal function parameters, with more pronounced effects observed in the high‐dose group. Histopathological analysis revealed that SDP effectively attenuated renal tubular dilation and reduced inflammatory cell infiltration. Mechanistically, SDP upregulated FXR expression while downregulating oxidative stress markers (gp91) and inflammatory mediators (TNF‐*α* and P‐MLK1) (Figure 5). Notably, using FXR knockout mice, we demonstrated that FXR is essential for SDP’s renoprotective effects, as its ablation significantly diminished the formula’s therapeutic efficacy. These findings not only confirm SDP’s ability to alleviate oxidative stress and inflammation in DN but also identify FXR as a crucial molecular target, with Yin Chen (*Artemisia capillaris*) likely being the key component mediating these effects. Further investigation into the FXR‐mediated mechanisms may provide novel therapeutic strategies for DN management.

## 5. Conclusion

This study provides robust evidence that SDP represents a mechanistically validated, multitarget therapy for DN, with FXR activation serving as its central mechanism of action. Through comprehensive clinical and experimental investigations, we have demonstrated that SDP achieves coordinated modulation of key pathological pathways in DN while maintaining an excellent safety profile. These findings not only support the therapeutic application of SDP but also open new avenues for developing integrative treatment strategies for diabetic kidney disease.

Furthermore, a multicenter clinical trial investigating SDP in DN is currently in progress, incorporating an extended follow‐up period of 3 months to assess long‐term efficacy and safety. Future studies should prioritize formulation optimization and exploration of synergistic drug combinations to further enhance clinical outcomes for DN patients.

**Nomenclature**
DN: diabetic nephropathy
SDP: Suyin Detoxification Prescription
TCM: traditional Chinese medicine
CKD: chronic kidney disease
FXR: farnesoid X receptor
eGFR: estimated glomerular filtration rate
Scr: serum creatinine
BUN: blood urea nitrogen
UA: uric acid
STZ: streptozotocin
HE: hematoxylin and eosin staining
PAS: periodic acid‐Schiff staining
WB: Western blot
TNF‐*α*
: tumor necrosis factor‐alpha
gp91: cytochrome
b‐245: heavy chain (NOX2)
P‐MLK1: phosphorylated mixed lineage kinase 1
OB: oral bioavailability
DL: drug‐likeness
GO: Gene Ontology
KEGG: Kyoto Encyclopedia of Genes and Genomes
DGLJ: dapagliflozin
SGLT2: sodium‐glucose cotransporter‐2

## Ethics Statement

This study complies with the ethical standards for trials and animal research and has been approved by the Ethics Committee of Pukou Hospital of Traditional Chinese Medicine (No. 2021NL‐182‐01, No. 20250008, and No. 20210028). The research conducted complies with the norms of clinical trials and animal experiments.

## Disclosure

We hereby declare that the preprint (doi:10.1101/2025.07.11.664291) is a prior work from our research group, which results in overlapping authorship. The research content and findings presented in this manuscript are entirely distinct [29]. We hereby declare that this original research manuscript, which has not been published previously nor is currently under consideration elsewhere (in whole or in part), represents the collaborative work of all authors. All authors have reviewed and approved the final version for submission.

## Conflicts of Interest

The authors declare no conflicts of interest.

## Author Contributions

Tuo Wei and Jiebo Huang conducted relevant clinical trials. Tuo Wei and Chao Shi conducted the molecular experiments and prepared the initial manuscript draft. Bingying Wan, Jing Cheng, Chen Yong, and Bei Huang provided experimental design guidance and chart preparation. Enchao Zhou and Yan Li conceived the study, supervised the research design and coordination, and contributed to manuscript preparation. Tuo Wei and Jiebo Huang contributed equally to this article.

## Funding

The study was funded by National Natural Science Foundation of China (10.13039/501100001809, 82474427), Traditional Chinese Medicine Science and Technology Research Project of Anhui Province (202303a0702001), Jiangsu Province Leading Talents Cultivation Project for Traditional Chinese Medicine (SLJ0319), and Li Yan National Famous Old Chinese Medicine Expert Studio, Teaching Letter of Chinese Traditional Medicine (2022) 75.

## Data Availability

The datasets generated during this study are available from the corresponding authors upon reasonable request, as they contain sensitive information that cannot be publicly shared due to ethical considerations.
